# Emergence of New Delhi metallo-beta-lactamase 1 and other carbapenemase-producing *Acinetobacter calcoaceticus-baumannii* complex among patients in hospitals in Ha Noi, Viet Nam

**DOI:** 10.1007/s10096-016-2784-8

**Published:** 2016-10-06

**Authors:** D. N. Tran, H. H. Tran, M. Matsui, M. Suzuki, S. Suzuki, K. Shibayama, T. D. Pham, T. T. Van Phuong, D. A. Dang, H. S. Trinh, C. T. Loan, L. T. V. Nga, H. R. van Doorn, H. F. L. Wertheim

**Affiliations:** 1 National Institute of Hygiene and Epidemiology, Yersin 1, Hanoi, Vietnam; 2 Department of Bacteriology II, National Institute of Infectious Diseases, Tokyo, Japan; 3 Viet-Duc Hospital, Hanoi, Vietnam; 4 Saint Paul Hospital, Hanoi, Vietnam; 5 Thanh Nhan Hospital, Hanoi, Vietnam; 6 Oxford University Clinical Research Unit, Hanoi, Vietnam; Nuffield Department of Clinical Medicine, Centre for Tropical Medicine, University of Oxford, Oxford, UK; 7 Department of Clinical Microbiology, Radboud UMC, Nijmegen, Netherlands

## Abstract

*Acinetobacter baumannii* is an important cause of multidrug-resistant hospital acquired infections in the world. Here, we investigate the presence of NDM-1 and other carbapenemases among carbapenem-resistant *A. baumannii* isolated between August 2010 and December 2014 from three large hospitals in Hanoi, Vietnam. We identified 23/582 isolates (4 %) (11 from hospital A, five from hospital B, and seven from hospital C) that were NDM-1 positive, and among them 18 carried additional carbapenemase genes, including seven isolates carrying NDM-1, IMP-1, and OXA-58 with high MICs for carbapenems. Genotyping indicated that NDM-1 carrying *A. baumannii* have expanded clonally in these hospitals. Five new STs (ST1135, ST1136, ST1137, ST1138, and ST1139) were identified. One isolate carried NDM-1 on a plasmid belonging to the N-repA replicon type; no NDM-1-positive plasmids were identified in the other isolates. We have shown the extent of the carbapenem resistance and the local clonal spread of *A. baumannii* carrying NDM-1 in these hospitals; coexistence of NDM-1 and IMP-1 is reported for the first time from Vietnam here, and this will further seriously limit future therapeutic options.

## Introduction

Antimicrobial resistance (AMR) has taken centre stage as a global health issue demanding public attention and commanding resources to understand where the international community will be placed in in 2020/2025 [1]. AMR is now deemed to be the biggest global threat facing humanity in the twenty-first century. The World Health Organisation recently published a mapping exercise on regional capacity, and South-East Asia lacked many basic reporting structures on AMR [2]. The problem of antibiotic resistance has recently reached a climax with the discovery of New Delhi metallo-beta-lactamase 1 (NDM-1) and MCR-1, plasmid-borne genes that confer resistance to the last-resort antibiotics carbapenems and colistin respectively [3, 4].

Many would argue that it was the advent of the NDM-1 metallo-beta-lactamase resistance mechanism appearing in Europe and being strongly linked to South Asia (mainly India and Pakistan) that became the universal game changer for the international importance of AMR [5]. It is now recognised that this is as much a public health issue (unabated dissemination in the community) as a hospital-based issue [6]. The NDM-1 gene encodes an enzyme that hydrolyses and inactivates all beta-lactam antibiotics including carbapenems, except for aztreonam, and thus induces resistance to carbapenems [3]. This gene has been identified on a variety of plasmids in a large number of Gram-negative pathogens and reported from most continents around the globe, especially from South-East Asia [6, 7].

*Acinetobacter baumannii* is an opportunistic pathogen in humans, and is an increasing cause of drug-resistant hospital-acquired infections across the world [8, 9]. *A. baumannii* is a strictly aerobic, non-motile, Gram-negative coccobacillus belonging to the *Acinetobacter calcoaceticus–baumannii* complex, within the family Moraxellaceae of the order Gammaproteobacteriae. In contrast to other *Acinetobacter* species, *A. baumannii* is more common in hospital environments and is capable of surviving on dry surfaces for months [10]. Resistance to carbapenems in *A. baumannii* is becoming common across all continents [9, 11]. The resistance is mainly mediated by OXA-type serine beta-lactamases, and IMP-type and VIM-type metallo-beta-lactamases [9].

NDM-1 now has been found in several different species of Enterobacteriaceae*. A. baumannii* carrying NDM-1 have been reported from clinical and environmental isolates in several countries [12–16].

In Vietnam, antibiotic resistance rates have been reportedly high. A combination of a high infectious diseases burden, unrestricted access to antibiotics, and poor infection control measures contribute to the emergence of antibiotic resistance in Vietnam [17, 18]. Enterobacteriaceae carrying NDM-1 have been reported from Vietnamese patients, healthy volunteers, and the environment [19–21]. *A. baumannii* is one of three (the others are *Pseudomonas aeruginosa* and *Klebsiella pneumoniae*) most common pathogens of hospital acquired infections (HAIs), accounting for 23.8 % of all HAIs causes in Vietnam, and almost 50 % of these were resistant to carbapenems [22]. The carbapenem resistance was caused by OXA-23 and was found in clinical and environmental isolates [7, 23]. Recently, NDM-1-producing *A. baumannii* was described from a hospital in Ho Chi Minh City in southern Vietnam [24]. Here, we collected isolates from three hospitals in Hanoi to detect carbapenem-resistant *A. baumannii* containing the NDM-1 gene and to determine the relationship of NDM-1-carrying isolates between these hospitals.

## Materials and methods

### Study sites

Between August 2010 and December 2014, we prospectively collected data and carbapenem-resistant *A. baumannii* isolates from three large hospitals: Saint Paul (A), Thanh Nhan (B) and Vietduc (C) in Hanoi, Vietnam.

Demographic and basic clinical information were collected from patients from whom carbapenem-resistant bacteria were cultured, including: age, gender, date of admission, clinical diagnosis, origin of collected sample, isolated bacterial strains, and date of sample collection. All of clinical isolates used in this study were obtained during standard patient care. Treatment and clinical outcome data were not available for this study. Isolates were sent to the National Institute of Hygiene and Epidemiology (NIHE) for further characterisation.

### Bacterial identification, susceptibility testing and detection of resistance genes

The microbiology laboratories of each hospital performed culture and species identification using commercial biochemical testing kits (API20E and API20NE, Biomérieux, Marcy l’Étoile, France). Minimum inhibitory concentrations (MICs) for carbapenem were performed by agar dilution according to CLSI 2012 guidelines and European Committee on Antimicrobial Susceptibility testing (EUCAST) breakpoints for colistin [25, 26]. The antibiotics used for MIC were imipenem (IMP), meropenem (MEM), cefotaxime (CTX), ceftazidime (CAZ), ciprofloxacin (CIP), and colistin (CS) (Sigma–Aldrich). The MBL E-test (Biomérieux) was used to screen for metallo-beta-lactamase production. NDM-1 was detected by polymerase chain reaction (PCR) using specific primers [3], other carbapenemases genes (KPC, IMP, VIM, SIM), and OXA genes as described elsewhere [27–30]. Resulting amplicons were sequenced using conventional sequencing.

### PFGE

Pulsed-field gel electrophoresis (PFGE) was carried following digestion with *Apa*I (Roche Diagnostic, Mannheim, Germany). *Salmonella braenderup* H9812 digested with *Xba*I served as reference molecular weight marker. DNA fragments were separated on a CHEF-DR III apparatus (Bio-Rad, Hercules, CA, USA) for 20 h at 6 V/cm at 14 °C with an initial pulse time of 0.5 s and final pulse time of 30s, as described elsewhere [5].

### Multilocus sequence typing (MLST)

MLST using the Oxford scheme was performed according to the protocol described on the MLST website (http://pubmlst.org/abaumannii/). Seven housekeeping genes were amplified by PCR, and sequenced and compared with the sequences submitted to the MLST database websites to determine Sequence Types (STs).

### Plasmid characterization

DNA plugs of the isolates harbouring *bla*_NDM-1_ were treated with restriction enzyme S1 (Invitrogen, Abingdon, UK) with *Salmonella braenderup* H9812 digested with *Xba*I as reference molecular weight marker and separated by PFGE. The Biometra–Analytik system (Jena, Germany) was used to transfer DNA fragments from the gel to nylon-membranes. Fragments were hybridised with NDM-1 probe labeled in HL-2000 HybriLinker (UPV, Germany). *Enterobacter cloacae* carrying the NDM-1 plasmid was used as positive control [7]. Plasmids hybridising with the NDM-1 probe were cut from the gel, purified, and typed as described elsewhere [31]

Conjugational transfer of NDM-1 plasmids to the laboratory strain *E. coli* J53 was done on Luria–Bertani broth without selection. After 16 h, the mixed culture was centrifuged, suspended in saline, and plated onto MacConkey agar containing sodium azide (100 mg/l) and meropenem (0.5 mg/l). Transconjugants were confirmed to have NDM-1 by PCR analysis. Plasmids were subsequently isolated and typed as described elsewhere [31].

### Statistical methods

PFGE data were analysed using BioNumerics version 6.5 (Applied Maths, USA). Isolates and patient data were analysed in MS Excel 2010 (Microsoft Corp., USA) using descriptive statistics as appropriate.

### Ethics statement

Ethical approval was obtained from the Ethical Committee of NIHE in 2010. All patient data were anonymised.

## Results

Between August 2010 and December 2014 1783 carbapenem-resistant Gram-negative bacteria were collected, 582 (32.6 %) of which were *A. baumannii. A baumannii* isolates were cultured from all three participating hospitals: 34 isolates in 2010, 220 in 2011, 279 in 2012, 47 in 2013, and two isolates in 2014. Fifty-five isolates were from hospital A, 106 from hospital B, and 421 from hospital C. Among these 582, *A. baumannii* isolates, 23 (4.0 %) were confirmed to be NDM-1-positive: 11 from hospital A, five from hospital B, and seven from hospital C (Table 2). In this study, we also investigated the presence of other carbapenem resistance genes. Among 559 carbapenem-resistant *A. baumannii* isolates negative to NDM-1, 550 (98.4 %) were positive for OXA-23, and two (0.35 %) isolates were positive for OXA-58. Two *A. baumannii* isolates were positive for IMP-1. The remaining *A. baumannii* isolates were negative for KPC, VIM, SIM, and OXA-48 genes (Table 1).

**Table 1:** Distribution of OXA and other carbapenemase genes in *A. baumannii* isolates negative to NDM-1 (*n* = 559)

Gene	OXA-23	OXA-24	OXA-58	IMP-1
Hospital				
A	44	0	0	0
B	96	0	0	1
C	410	0	2	1
Total	550	0	2	2

The median age of the patients with NDM-1-positive isolates was 26 (range: 1 to 77 years). Eight patients were under 1 year of age. Among NDM-1-positive patients, 20 were male and three were female. Seventeen strains were isolated from endotracheal aspirates, three from blood, two from urine and one from pus.

Interestingly, 18/23 NDM-1-producing *A. baumannii* carried additional carbapenemase genes: NDM-1, IMP-1, and OXA-58 were found in seven isolates from 2010–2011 in hospital A. One isolate in hospital B in 2011 was positive for NDM-1, OXA-23, and OXA-58. Eight isolates were positive for NDM-1 and OXA-58 (three in hospital A, one in hospital B, and four in hospital C), and two isolates were carrying both NDM-1 and OXA-23 in hospital A (2014) and C (2013) (Table 2).

**Table 2:** Characterisation of NDM-1 carrying *A. baumannii* isolates

No	Hospital	Year	Isolate source	Depart	Carbapenemase genes				MIC (mg/l)						MLST	PFGE
					NDM-1	IMP-1	OXA-23	OXA-58	IPM	MEM	CIP	CAZ	CTX	CS		
320	B	2011	Bronchial fluid	ICU	+	−	−	−	64	64	256	>512	>512	1	ST302	II
821	C	2012	Urine	Urology	+	−	−	−	32	32	0.25	>512	>512	2	ST1138	−
1105	B	2013	Blood	ICU	+	−	−	−	16	16	64	>512	256	0,25	ST861	I
1146	B	2013	Blood	ICU	+	−	−	−	32	16	64	>512	256	0.25	ST861	I
1191	C	2013	Bronchial fluid	ICU	+	−	−	−	32	32	4	>512	>512	0.25	ST1097 (CC92)	−
65	A	2010	Bronchial fluid	ICU	+	+	−	+	128	64	0.0625	>512	>512	2	ST1261	−
271	A	2011	Bronchial fluid	Pediatric	+	+	−	+	256	128	0.25	>512	>512	1	ST1135	III
275	A	2011	Bronchial fluid	Pediatric	+	+	−	+	256	128	0.25	>512	>512	2	ST1135	III
327	A	2011	Bronchial fluid	Pediatric	+	+	−	+	256	64	0.5	>512	>512	1	ST1135	III
282	A	2011	Bronchial fluid	Pediatric	+	+	−	+	256	64	0.125	>512	>512	2	ST1136	III
351	A	2011	Bronchial fluid	Pediatric	+	+	−	+	256	128	0.5	>512	>512	2	ST1135	III
357	A	2011	Bronchial fluid	Pediatric	+	+	−	+	256	128	0.5	>512	>512	2	ST1135	III
340	B	2011	Bronchial fluid	ICU	+	−	+	+	16	16	128	>512	64	1	ST91	−
303	C	2011	Bronchial fluid	ICU	+	−	−	+	128	64	128	>512	>512	**1**	ST302	II
393	A	2011	Bronchial fluid	Pediatric	+	−	−	+	32	16	0.25	>512	>512	1	ST1137	III
650	A	2012	Bronchial fluid	Pediatric	+	−	−	+	64	128	0.25	>512	>512	2	ST1135	III
856	C	2012	Bronchial fluid	ICU	+	−	−	+	64	64	8	>512	>512	2	ST1139 (CC92)	−
947	A	2012	Bronchial fluid	Pediatric	+	−	−	+	32	16	0.5	>512	256	0.25	ST655	−
1057	C	2012	Urine	Urology	+	−	−	+	16	32	64	>512	>512	0.25	ST593	−
1398	B	2013	Blood	Hemodialysis	+	−	−	+	2	0,5	0.5	32	64	0.25	ST861	I
1267	C	2013	Bronchial fluid	ICU	+	−	−	+	16	16	16	>512	256	0.25	ST208 (CC92)	IV
1413	C	2013	Pus	Infectious surgery	+	−	+	−	16	16	0.25	>512	>512	0.25	ST208 (CC92)	IV
1539	A	2014	Bronchial Fluid	ICU	+	−	+	−	8	8	64	>512	128	0.25	ST493 (CC92)	−

All carbapenem-resistant isolates were found to be susceptible to colistin, and 13/23 isolates (57 %) remained susceptible to ciprofloxacin. All isolates were resistant to ceftriaxone and ceftazidime. For carbapenems, one *A. baumannii* isolate from blood in hospital B was susceptible to meropenem (0.5 mg/l) but had reduced susceptibility to imipenem (2 mg/l), and 22/23 isolates were resistant to both imipenem and meropenem (8–256 mg/l). A significantly higher level of carbapenem resistance was detected in 7 *A. baumannii* with co-existent NDM-1, IMP-1, and OXA-58 (MIC imipenem = 128-256 mg/l, median 256, and MIC meropenem = 64–128 mg/l, median 128) when compared with other NDM-1-producing *A. baumannii* (MIC = 8–128 mg/l for both imipenem and meropenem, median 32 and 16 respectively, *p* = 0.001) (Table 2).

**Fig. 1:**
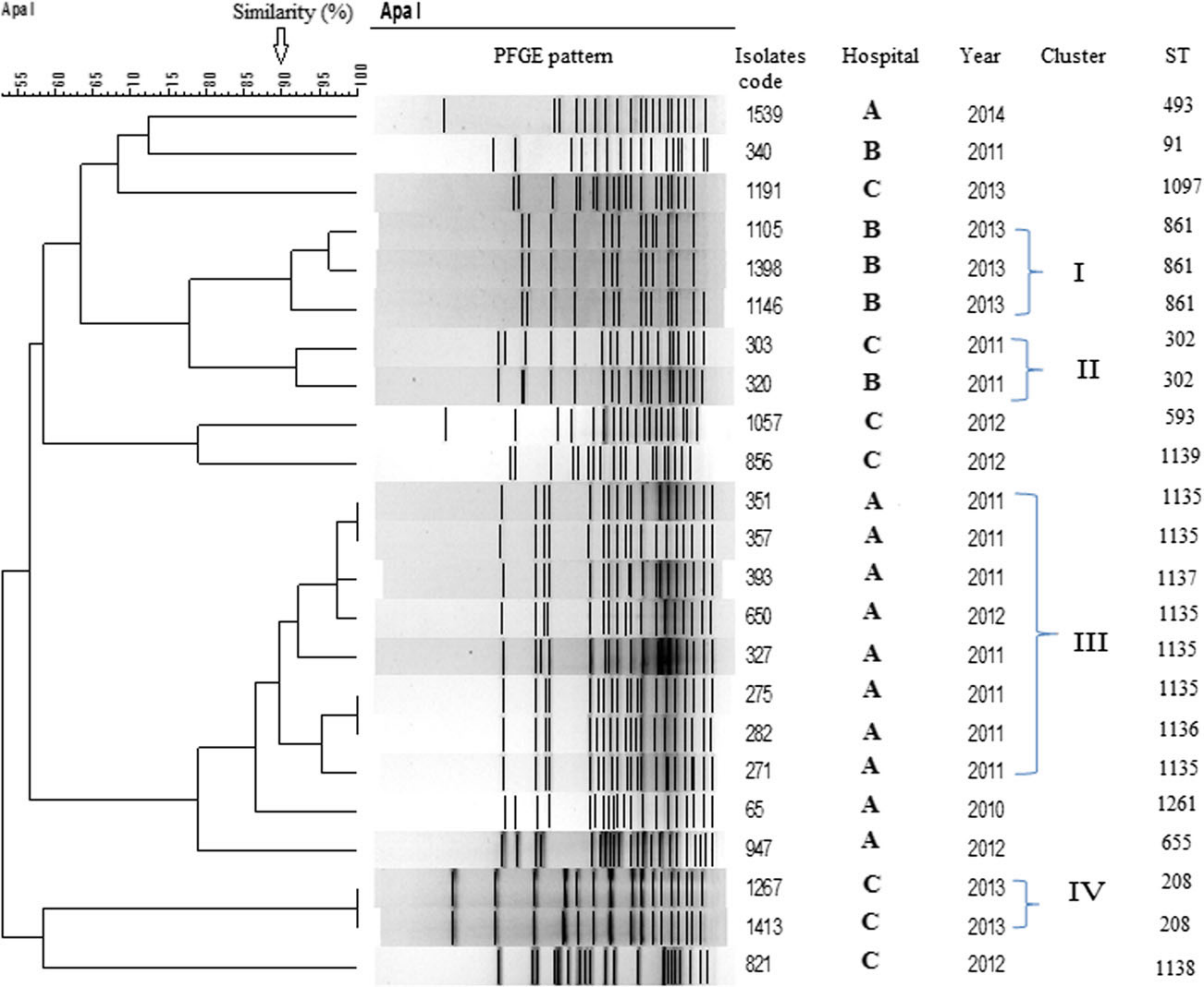
PFGE and MLST analysis of 23 isolates of NDM-1–positive *A. baumannii*. Four clusters (*I*–*IV*) with more than 90 % similarity were identified

In PFGE analysis of the 23 *bla*_NDM-1_-positive isolates recovered in this study, four PFGE clusters were observed (Fig. 1). Cluster I contains three isolates from hospital B in 2013, cluster II contains two isolates in 2011 from hospital B and C, cluster III contains eight isolates (seven in 2011 and one isolate in 2012) in hospital A, and cluster IV contains two isolates from hospital C. These results indicate that NDM-1-carrying *A. baumannii* has expanded clonally in these hospitals and has the potential for intra-hospital patient-to-patient spread.

The *A. baumannii* isolates producing NDM-1 belonged to ST91, ST208, ST302, ST493, ST593, ST655, ST861, ST1097, ST1261, and to five new STs: ST1135, ST1136, ST1137, ST1138, and ST1139. ST1139 belongs to international clone CC92, and was found in the ICU of hospital C in 2010 (Fig. 1 and Table 2). Six of the *A. baumannii* isolates producing NDM-1 from bronchial fluid of patients in hospital A belonged to the new ST1135, and two new ST302 were found in hospital B and C. We have also analysed a selection (8/559) of carbapenem-resistant *A. baumannii* negative to NDM-1, and these isolates belonged to ST91, ST92, ST109, ST195, ST495, ST620, and ST1140.

**Fig. 2:**
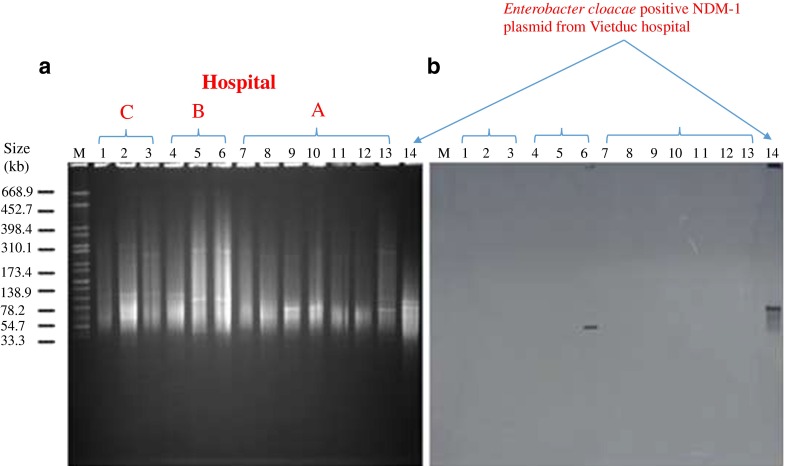
S1-PFGE and Southern blotting of plasmids carrying NDM-1 from clinical isolates. Pulse-field gel of S1 nuclease-treated plasmid DNA of selected *A. baumannii* from clinical isolates in three hospitals, stained with ethidium bromide. The molecular weight marker is *Salmonella braenderup* H9812 cut with *Xba*I (**a**). Autodiagram of gel A showing plasmids carrying NDM-1 (**b**). *Enterobacter cloacae* positive NDM-1 plasmid from Vietduc hospital

Southern blotting only detected NDM-1 in one plasmid belonging to the N-repA replicon type, isolated from *A. baumannii* strain 1146 from blood of a patient in the ICU of hospital B (Fig. 2). Conjugation assay was performed, but we found no transconjugant strains. There was lack of plasmid in 22/23 positive NDM-1, suggesting that spread is mainly clonal and not through horizontal gene transfer.

## Discussion

Since the first description of NDM-1 [3], the rapid dissemination of this gene among Enterobacteriaceae has been reported from many countries around the world [5, 6]. However, NDM-1-production has also been found in other Gram-negative bacteria, such as *A. baumannii* [12–16]. In Vietnam, NDM-1-producing Enterobacteriaceae were commonly isolated from patients admitted to a Vietnamese surgical hospital, and from the environment [19, 20], and recently two NDM-1 producing *A. baumannii* were described from a hospital in Ho Chi Minh City in southern Vietnam [24]. NDM genes have been described in *A. baumannii* from other countries from 2010 onwards, and NDM associated with the *Tn*125 transposon has been hypothesised to originate from *A. baumannii* isolates in North Africa prior to transfer to Enterobacteriaceae [8, 32].

Here, we report for the first time the isolation of NDM-1-producing *A. baumannii* from patients in northern Vietnam in 23/582 isolates (4 %) collected between 2010 and 2014, with the first isolate being detected in 2010. As is commonly found in other countries, the majority of NDM-1 was not detected on plasmids and presumed to be integron-associated or chromosomal [33]. Also, NDM-1 was found to co-exist with other carbapenemases including IMP-1 reported in Vietnam for the first time, and OXA-58. The contribution of either of these genes to the carbapenem resistance is unclear, but MIC of isolates co-carrying IMP-1 was significantly higher.

Low level carbapenem resistance in *A. baumannii* is often mediated through OXA-51 like enzymes belonging to the class D ß-lactamase family, which exhibit weak carbapenemase activity and are labeled CHDL for carbapenem-hydrolysing class D ß-lactamases. OXA-23 is the most commonly identified CHDL world-wide in *A. baumannii*, and was also found in 550/559 NDM-1-negative carbapenem-resistant isolates and 3/23 NDM-1 positive isolates. OXA-58 was detected in 2/559 NDM-1-negative and 16/23 NDM-1-positive isolates. True carbapenemase activity (metallo-ß-lactamases, class B ß-lactamases) in *A. baumannii* can be mediated by IMP, VIM, SIM-1, NDM, and other genes. Here, we only detected IMP-1 in 2/550 NDM-1-negative and 7/23 NDM-1-positive isolates. This distribution of resistance genes suggests different phylogenies of the NDM-1-positive and -negative isolates, which was confirmed by MLST. There was limited overlap with STs described in a large Asian surveillance project [34]. MLST further showed several new sequence types and clustering of isolates suggestive of local evolution, clonal expansion, and within-hospital spread.

This study shows the proportion of carbapenem-resistant *A. baumannii* isolates carrying carbapenemase gene in three large hospitals in Ha Noi. There are limitations to the results of this study that are caused by the limited amount of metadata that were collected, and by the fact that we only analysed carbapenem-resistant isolates that were found in the laboratory among samples sent in to the laboratory by treating physicians. Therefore, we cannot draw conclusions whether isolates were likely to be community- or hospital-acquired, and whether they were causing illness or commensal, whether clustering is due to the hospital environment serving as a reservoir or patient-to-patient transmission, what the percentage of total isolates of *A. baumannii* with carbapenem resistance is, and other epidemiologic analyses requiring hospital and laboratory denominators.

## Conclusion

In this study, we show that NDM-1 has already been present in *A. baumannii* in northern Vietnam since 2010. The extent of the carbapenem resistance and the local clonal spread of *A. baumannii* carrying NDM-1 in these hospitals, as well as coexistence of NDM-1 and IMP-1, is reported for the first time from Vietnam here, and this will further seriously limit future therapeutic options.
